# Sequencing Cellular Therapies in the Management of Follicular Lymphoma

**DOI:** 10.3390/cells14211671

**Published:** 2025-10-25

**Authors:** Ádám Jóna, Árpád Illés

**Affiliations:** 1 Department of Hematology, Faculty of Medicine, University of Debrecen, Member of the ERN-EuroBloodNet (European Reference Network on Rare Haematological Diseases), H-4032 Debrecen, Hungary; illesarpaddr@gmail.com; 2 Doctoral School of Clinical Medicine, University of Debrecen, H-4032 Debrecen, Hungary

**Keywords:** follicular lymphoma, cellular therapies, CAR T-cell therapy, bispecific antibodies, stem cell transplantation

## Abstract

**Highlights:**

**What are the main findings?**

1. This article thoroughly reviews the current landscape and optimal sequencing of advanced cellular therapies, including autologous stem cell transplantation, allogeneic stem cell transplantation, and CAR T-cell therapy, for managing relapsed/refractory follicular lymphoma.
2. It highlights the significant efficacy of CAR T-cell therapy in achieving high response rates and durable remissions, potentially serving as a bridge between transplant modalities, and discusses the role of newer bispecific antibodies as convenient off-the-shelf options.

**What is the implication of the main finding?**

3. The evolving treatment paradigm for follicular lymphoma necessitates highly individualized decisions that consider patient characteristics, disease features, and the complex interplay of various cellular and novel immunotherapies.
4. Further research into predictive biomarkers, refined treatment algorithms, and the impact of sequential therapies (e.g., bispecific antibodies before CAR-T) is crucial to optimizing patient outcomes and integrating these powerful new options effectively into clinical practice.

**Abstract:**

Follicular lymphoma management is rapidly evolving with advanced cellular therapies. This review examines the optimal sequencing of autologous stem cell transplantation (autoSCT), allogeneic stem cell transplantation (alloSCT), and CAR T-cell therapy. AutoSCT is a crucial intervention for chemosensitive relapsed FL, prolonging progression-free survival, though not typically curative. AlloSCT, offering a potential cure via a graft-versus-lymphoma effect, carries significant risks like graft-versus-host disease and non-relapse mortality, thus primarily serving as a salvage option for high-risk or treatment-refractory cases after other modalities, including autoSCT. CAR T-cell therapy, utilizing genetically modified T cells targeting CD19, has revolutionized relapsed/refractory FL. Products like axicabtagene ciloleucel, tisagenlecleucel, and lisocabtagene maraleucel have demonstrated high response rates and durable remissions even in heavily pretreated patients with high-risk features. This potent therapy is increasingly considered a bridge between autoSCT and alloSCT, expanding treatment options. Additionally, bispecific antibodies such as mosunetuzumab, epcoritamab and odrenextamab provide convenient off-the-shelf options, exhibiting strong efficacy and favorable safety. However, their impact on subsequent CAR-T outcomes, especially with CD19-targeting bispecifics, remains an area of ongoing investigation and uncertainty. The complex interplay of these therapies necessitates individualized decisions, emphasizing patient characteristics and disease-specific factors to optimize outcomes in FL. Further research into predictive biomarkers and refined treatment algorithms is crucial for future management.

## 1. Introduction

Follicular lymphoma (FL) is the second most prevalent non-Hodgkin lymphoma globally. It commonly affects middle-aged to older adults and is often diagnosed at advanced stages. FL originates from germinal center B cells, marked by the t(14;18) translocation that leads to BCL2 overexpression, inhibiting programmed cell death. Additional mutations in epigenetic regulators like CREBBP, EZH2, and KMT2D contribute to disease heterogeneity. Clinically, FL varies from slow progression to aggressive transformation, impacting treatment outcomes. The tumor microenvironment also influences disease progression and therapy response. Large-scale registries and standardized definitions are crucial for understanding FL’s complexity and improving management [1].

FL is characterized by an indolent course, yet a subset of patients experiences early progression or transformation to more aggressive histologies, such as diffuse large B-cell lymphoma or Burkitt lymphoma, which is referred to as histologic transformation (HT) [2,3,4]. The risk of HT and recurrence shapes the therapeutic approach, as these events are associated with poorer outcomes and influence subsequent treatment decisions. The current treatment landscape for FL is multifaceted, integrating conventional chemoimmunotherapy, cellular therapies, and novel immunotherapeutic agents. First-line therapy typically involves anti-CD20 monoclonal antibodies, often in combination with chemotherapy, which has led to substantial improvements in progression-free and overall survival. However, most patients will eventually relapse, necessitating further lines of therapy. For those with relapsed or refractory (R/R) disease, particularly after two or more prior lines of systemic therapy, the therapeutic paradigm becomes increasingly complex [5,6].

The treatment landscape for relapsed or refractory follicular lymphoma (FL) after second-line therapy has expanded significantly with the introduction of several novel agents and therapeutic strategies. The choice of treatment is highly individualized and depends on factors such as prior therapies, duration of remission, patient fitness, and disease characteristics. This includes T-cell modulators like bispecific antibodies and CAR-T cell therapy, EZH2 inhibitor tazemetostat, and BTK inhibitors. Clinical trials are also actively investigating new combinations and emerging therapies [7,8,9].

This review aims to address the positioning of cellular therapies, specifically autologous stem cell transplantation, allogeneic stem cell transplantation, the use of bispecific antibodies and CAR-T cell therapy, in the treatment algorithm for FL. We will analyze the role of each therapy, discuss its sequencing, and consider the impact of novel agents like bispecific antibodies on cellular therapy outcomes. We will also address the challenges in sequencing these therapies, as well as the importance of considering individual patient characteristics and disease-specific factors [10].

## 2. The Importance of Autologous Stem Cell Transplantation

Autologous stem cell transplantation (autoSCT) has been a crucial intervention for relapsed follicular lymphoma [11,12]. In this procedure, a patient’s stem cells are collected and stored. High-dose chemotherapy, typically with agents like BEAM (carmustine, etoposide, cytarabine, and melphalan), is administered, followed by reinfusion of the previously collected stem cells to restore bone marrow function [12].

Consolidation with autoSCT as front-line treatment in FL did not show any improvement in OS [11]. AutoSCT is primarily used for patients who have relapsed after initial treatment but whose lymphoma remains sensitive to chemotherapy (e.g., R-DHAP, R-ICE) [12]. It has been shown to prolong progression-free survival in these patients [12]. A median PFS of over 4 years compared to approximately 1 year for the standard chemotherapy arm was shown in the pre-rituximab era. Nowadays, a 5-year PFS still ranges from 40 to 66% with PFS curves suggesting a plateau, indicating a potential for cure in a subset of patients. However, autoSCT is not generally considered curative for most patients, as relapses can still occur, but long-term remissions are also present.

Traditionally, autoSCT has been considered for younger and fit individuals, particularly those who experience early relapse or transformation of FL, as these patients tend to have a more aggressive disease course and may derive greater benefit from intensive therapy. Early intervention with autoSCT, particularly in patients with high-risk features such as progression of disease within 24 months (POD24) or double-refractory disease, may improve outcomes, although these patients still face inferior responses compared to those without such risk factors [13,14,15,16].

The role of autoSCT has been debated in the era of novel therapies, but it remains a standard of care for many patients with chemosensitive relapsed FL. The procedure is well-established, and the risks are relatively well-defined. Important factors in determining the suitability of autoSCT include the patient’s age, performance status, chemosensitivity, and prior treatment history.

While autoSCT provides a period of remission, the duration of remission varies. Some patients experience long-term disease control, while others relapse relatively quickly. Factors associated with shorter remissions include high-risk disease features, such as a high Follicular Lymphoma International Prognostic Index score, early relapse, and bulky disease [17].

The sequencing of autoSCT relative to other cellular therapies, such as chimeric antigen receptor (CAR) T-cell therapy and allogeneic hematopoietic cell transplantation (alloSCT), is an area of active investigation and debate. The approval and increasing use of CAR T-cell therapies have expanded the therapeutic landscape for relapsed/refractory FL, yet have also introduced uncertainty regarding the optimal timing and sequencing of auto-HCT in relation to these novel modalities [11]. Notably, patients who have recently undergone autoSCT are often excluded from CAR T-cell therapy trials, reflecting concerns about overlapping toxicities and the impact of prior intensive therapies on subsequent cellular therapy outcomes. Despite these uncertainties, autoSCT continues to be considered a preferred initial cellular therapy option for selected patients with relapsed FL, particularly those with chemosensitive disease and adequate performance status [18]. The emergence of bispecific antibodies and their integration into treatment algorithms further complicates the sequencing of autoSCT, as the impact of prior or subsequent use of these agents on transplant outcomes remains to be fully elucidated. The interplay between patient selection, timing of transplantation, and the integration of novel agents will continue to shape the use of auto-HCT in FL management.

## 3. Allogeneic Stem Cell Transplantation: A Curative Option?

Allogeneic stem cell transplantation (alloSCT) stands as a pivotal cellular therapy for certain hematological malignancies, including FL. This complex procedure involves transplanting hematopoietic stem cells from a healthy donor into a patient, aiming to replace the diseased bone marrow and immune system. A key mechanism underpinning its therapeutic potential is the graft-versus-lymphoma effect (GVL), where immune cells from the donor recognize and eliminate residual lymphoma cells in the recipient [18,19]. This donor-derived immune response is crucial for achieving durable long-term disease control, particularly in cases of relapse after autologous transplant [18].

There are primarily two types of conditioning regimens employed before alloSCT: myeloablative (MAC) and reduced-intensity conditioning (RIC). MAC alloSCT involves high-dose chemotherapy, sometimes combined with radiation, designed to completely eradicate the patient’s bone marrow. This aggressive approach offers the possibility of a cure for FL, backed by compelling evidence of a strong GVL [19]. However, the intensity of MAC regimens is associated with significant toxicity. In contrast, RIC alloSCT has emerged as a less toxic alternative [18], making it accessible to a broader range of patients, including those who are older or less physically fit and might not tolerate a full myeloablative regimen [20]. While RIC aims to suppress the patient’s immune system sufficiently to allow engraftment of donor cells and facilitate the GVL, it generally carries a lower immediate toxicity burden. Despite its advantages, alloSCT, regardless of the conditioning regimen, is notably associated with non-relapse mortality, which refers to deaths not directly caused by the original disease [11]. Comparative studies between autoSCT and alloSCT indicate that autoSCT is associated with a lower rate of non-relapse mortality (NRM); however, this benefit is often offset by a higher risk of disease relapse in the autoSCT group [11]. NRM has been reported from 15% to 30% at 5 years. The adoption of RIC regimens has been key to lowering the NRM (8–17%) compared to older, myeloablative regimens, which were associated with NRM rates as high as 30% to 40%.

Despite its curative potential, alloSCT carries substantial risks that necessitate careful patient selection. These risks include graft-versus-host disease (GVHD), a serious complication where donor immune cells attack healthy recipient tissues, and the aforementioned non-relapse mortality [19,21]. GVHD occurrence varies depending on the conditioning regimen, GVHD prophylaxis, donor type, and other factors. Acute GVHD often falls in the range of 11% to 30% at 100 days post-transplant. Chronic GVHD rates can be higher, often ranging from approximately 32% to over 60% at 2 years or later. Consequently, alloSCT is generally reserved for patients who have relapsed after initial treatments, such as autoSCT, or those presenting with high-risk disease features that predict poor outcomes with less intensive therapies [19]. The decision to proceed with alloSCT is a complex one, requiring thorough consideration of the potential benefits (long-term remission or cure) against the significant risks, as well as the patient’s overall health status and the availability of a suitable and the measure of donor match.

It is critical to note that alloSCT is not typically considered a primary cellular therapy in the management of FL. Instead, its role is largely defined as a salvage therapy—a treatment option pursued after the failure of other established treatments, including autologous stem cell transplantation and, more recently, CAR-T cell therapy [22]. The inherent risks associated with alloSCT, particularly GVHD, make it a less attractive choice as an initial therapeutic approach compared to the much safer autoSCT or the increasingly available CAR-T cell therapies. Its positioning later in the treatment algorithm reflects its potent, yet risk-laden, ability to induce a strong graft-versus-lymphoma effect in patients with otherwise refractory or relapsed disease.

## 4. CAR-T Cell Therapy: A Bridge Between Transplants?

CAR-T cell therapy has revolutionized the treatment of relapsed/refractory FL [23]. This approach involves genetically modifying a patient’s T cells to express a chimeric antigen receptor that targets a specific protein on lymphoma cells, such as CD19 [22]. These modified T cells are then infused back into the patient to eliminate lymphoma cells. These therapies have demonstrated high response rates and durable remissions in heavily pretreated patients. The approved agents have been shown to be effective in their respective clinical trials [24].

Several CAR-T cell products have been approved for R/R FL, including axicabtagene ciloleucel, tisagenlecleucel and lisocabtagene maraleucel [25,26,27]. A collaborative effort by the American Society for Transplantation and Cellular Therapy and the European Society for Blood and Marrow Transplantation recommended CAR-T as a treatment option for patients with POD24 who did not achieve complete or partial remission after second or subsequent line therapies [11]. Furthermore, the panel recommends CAR-T for relapsed FL patients who have relapsed after allo-HCT and are either untreated or did not achieve complete or partial remission to their most recent anti-lymphoma treatment.

### 4.1. Major CAR-T Trials in Follicular Lymphoma

The ZUMA-5 study was a phase 2, multicenter, single-arm trial that evaluated the efficacy and safety of axicabtagene ciloleucel (axi-cel), an autologous anti-CD19 CAR T-cell therapy, in patients with relapsed/refractory (R/R) indolent non-Hodgkin lymphoma (iNHL), including FL and marginal zone lymphoma (MZL). Patients in the study had received at least two prior lines of systemic therapy, including an anti-CD20 monoclonal antibody combined with an alkylating agent [25]. After a median follow-up of 41.7 months for FL patients and 31.8 months for MZL patients, axi-cel demonstrated durable responses with very few relapses beyond two years. Key findings from the ZUMA-5 study include: overall Response Rate (ORR) was 94% in FL and 77% in MZL. The primary analysis reported a 74% complete response rate. Median PFS was 40.2 months in FL and was not reached in MZL. Medians of overall survival were not reached in either disease type. The study showed a manageable safety profile. Elevated baseline total metabolic tumor volume and recent prior bendamustine use were found to negatively correlate with clinical and pharmacokinetic outcomes, potentially affecting durable remissions in FL patients.

The ELARA study was a phase 2, single-arm, global, multicenter, open-label trial designed to investigate the efficacy and safety of tisagenlecleucel in adult patients with R/R FL (grades 1–3A). Eligible patients had received at least two prior lines of therapy, including an anti-CD20 antibody and an alkylating agent, or had relapsed after autologous stem cell transplantation. The trial included patients with high-risk features such as progression of disease within 24 months from first immunochemotherapy and high baseline tumor burden, underscoring its relevance for a challenging patient population [26]. With a median follow-up of 28.9 months, tisagenlecleucel demonstrated highly durable efficacy and a favorable safety profile. The study reported an overall response rate of 86% and a complete response rate of 69% Furthermore, the estimated 24-month progression-free survival rate was 57.4%, the 24-month duration of response was 66.4%, and the 24-month overall survival rate was 87.7%. The median progression-free survival, duration of response, and overall survival were not reached, suggesting sustained benefit. Importantly, the long-term efficacy observed extended to patients with high metabolic tumor volume, bulky disease, double refractory disease, and high FLIPI scores, supporting the broad applicability of tisagenlecleucel. In terms of safety, the profile of tisagenlecleucel remained consistent with previous reports, with no new safety signals or treatment-related deaths observed in this extended follow-up. Grade ≥3 cytokine release syndrome and immune effector cell–associated neurotoxicity syndrome occurred in a very low percentage of patients (≤1%). While some patients experienced infections (16.5% any grade, 9.3% Grade ≥ 3) and cytopenias, the majority of cytopenias resolved over time. Exploratory biomarker analyses revealed that low levels of tumor-infiltrating LAG3 + CD3+ exhausted T cells and higher baseline levels of naive CD8+ T cells were significantly associated with improved outcomes.

The TRANSCEND FL study [27] was a phase 2, global, multicenter, open-label, single-arm, multi-cohort study that investigated lisocabtagene maraleucel (liso-cel), an autologous, CD19-directed CAR T-cell product, in adult patients with relapsed/refractory (R/R) follicular lymphoma. The study population included both patients who had received three or more prior lines of therapy (3L+) and, notably, patients in the second-line (2L) setting who all had progression of disease within 24 months from their initial immunochemotherapy. This marks it as the first study to report CAR T-cell therapy outcomes in 2L R/R FL patients with high-risk features [27]. A total of 130 patients received liso-cel, with a median follow-up of 18.9 months. The study demonstrated high efficacy across both cohorts. In the third-line or later FL group (*n* = 101), the overall response rate was 97%, with a complete remission rate of 94%. For the second-line FL group (*n* = 23), the ORR was 96%, and all responders achieved a complete remission. Responses were rapid and durable, and efficacy remained consistent across various high-risk subgroups, including those with progression of disease within 24 months from initial immunochemotherapy, double-refractory disease, and high tumor burden.

Regarding safety, lisocabtagene maraleucel exhibited a manageable profile. Cytokine release syndrome occurred in 58% of patients, with Grade ≥3 events in 1%. Neurological events were observed in 15% of patients, with Grade ≥3 events in 2%. The most common Grade ≥3 treatment-emergent adverse events were cytopenias, particularly neutropenia. The median age of the study population was relatively young, consistent with other phase 2 CAR T-cell studies for FL. Overall, TRANSCEND FL demonstrated significant benefit for patients with R/R FL, including those with challenging high-risk characteristics.

In comparison, all three trials consistently show the significant efficacy of CAR-T cell therapy in relapsed/refractory follicular lymphoma, evidenced by high overall response and complete remission rates. (Table 1) TRANSCEND FL reported slightly higher response and complete remission rates, particularly notable for its strong outcomes in second-line patients [27]. ZUMA-5 also demonstrated robust and durable responses in a broader iNHL population [25]. ELARA showcased sustained efficacy and a generally favorable safety profile [26]. Collectively, these studies underscore the transformative impact of CAR-T cell therapy as a crucial and highly effective treatment option in the evolving landscape of follicular lymphoma management.

### 4.2. CAR-T Between AutoSCT and AlloSCT

CAR-T cell therapy is emerging as a potential bridge between autoSCT and alloSCT. It offers a treatment option for patients who have failed autoSCT but are not ideal candidates for alloSCT due to age, comorbidities, or lack of a suitable donor [19]. The role of CAR-T expands the armamentarium of effective treatments for patients with R/R FL but also generates uncertainty regarding the optimal timing and sequencing of cellular therapies and autologous and allogeneic HCT in FL [11]. In some areas CAR-T may replace autoSCT, particularly as patients may not be fit for autoSCT but are for CAR-T. Some patients may not lead to alloSCT as many never fit for it.

## 5. The Role of Bispecific Antibodies

Bispecific antibodies represent another class of immunotherapies for FL, functioning as T-cell modulators that redirect T cells to lymphoma cells. They bind to two different targets, typically a tumor-associated antigen (e.g., CD20) and a T-cell receptor (e.g., CD3), bridging the lymphoma cell and the T cell to facilitate T-cell-mediated killing [23,28]. Bispecific antibodies offer off-the-shelf convenience and might be more easily accessible [24] (Table 2).

A multicenter study phase 2, single-arm, evaluating the fixed-duration mosunetuzumab, a CD20 × CD3 T-cell-engaging bispecific monoclonal antibody, in patients with relapsed or refractory follicular lymphoma. The study included patients aged 18 years or older with histologically confirmed follicular lymphoma (grade 1–3a) and an ECOG performance status of 0–1, who had disease relapsed or refractory to two or more previous lines of treatment, including an anti-CD20 therapy and an alkylating agent [29]. Mosunetuzumab was administered as step-up dosing at treatment initiation, and patients with a complete response completed treatment after cycle 8, whereas patients with a partial response or stable disease continued treatment for up to 17 cycles.

With a median follow-up of 18.3 months, mosunetuzumab demonstrated high complete response rates and durable remissions in this heavily pretreated population, including those with high-risk disease. The study met its primary efficacy endpoint, with a complete response rate of 60%. Responses were observed to be early and durable, and consistent across various patient subgroups.

Regarding safety, mosunetuzumab exhibited a favorable profile, allowing for potential outpatient administration. The most common adverse event was cytokine release syndrome, occurring in 44% of patients, predominantly grade 1–2 (42%), with grade 3 in 1% and grade 4 in 1%. Step-up dosing effectively mitigated CRS. Overall, the study concluded that fixed-duration mosunetuzumab is an active and well-tolerated treatment option for patients with relapsed or refractory follicular lymphoma.

The three-year follow-up analysis of mosunetuzumab in relapsed or refractory follicular lymphoma significantly extended the observation period beyond the initial trial [30]. While the pivotal trial [29] had a median follow-up of 18.3 months, the follow-up [30] now reports a median follow-up of 37.4 months. This longer follow-up shows that the high rate of durable responses observed initially is sustained, with a significant proportion of patients remaining free from progression at three years. Specifically, for patients who achieved a complete response, the median progression-free survival was 37.3 months, and the 36-month overall survival event-free rate was 94.5%. This extended data reinforces the long-term efficacy and manageable safety profile of fixed-duration mosunetuzumab, including the gradual recovery of B-cell counts after treatment, with a median time to recovery of 18.4 months for those with complete responses.

Epcoritamab is another bispecific antibody approved for use in relapsed/refractory FL [24]. The EPCORE NHL-1 study was a phase 1–2, multicenter, single-arm trial investigating epcoritamab, a CD3 × CD20 bispecific antibody, in patients with multiply relapsed or refractory follicular lymphoma. The study included patients aged 18 years or older with relapsed or refractory CD20+ follicular lymphoma (grade 1–3A) who had received at least two prior lines of therapy.

Epcoritamab was administered subcutaneously using a step-up dosing regimen in cycle 1 to manage cytokine release syndrome. An optimized three-stage step-up dosing regimen, along with prophylactic dexamethasone and adequate hydration, was found to reduce CRS rates. Epcoritamab was given until disease progression or unacceptable toxicity. The trial demonstrated robust and clinically meaningful efficacy with epcoritamab monotherapy in heavily pretreated patients, including those with high-risk disease. High rates of measurable residual disease negativity were observed and were associated with improved progression-free survival.

The safety profile of epcoritamab was manageable. The most common grade 3–4 adverse event was neutropenia. In the pivotal cohort, Grade 1–2 CRS occurred in 65% of patients, with Grade 3 CRS in 2%. Immune effector cell-associated neurotoxicity syndrome was reported in 6% of patients in the pivotal cohort, mostly Grade 1 or 2. Notably, in the cycle 1 optimization cohort, the incidence of CRS was reduced to 49%, with no Grade 3 or worse events, and no ICANS was reported. Overall, EPCORE NHL-1 indicates that epcoritamab has the potential to be an important treatment option for relapsed or refractory follicular lymphoma.

At the 3-year follow-up, epcoritamab demonstrated sustained and durable response rates without any novel safety issues found [31].

Odronextamab, which is another off-the-shelf, human CD20 × CD3 bispecific antibody, is being evaluated in patients with R/R FL from the ELM-2 phase II study and is currently (August 2025) under review by the US Food and Drug Administration. This open-label, multicenter study enrolled 128 patients with R/R follicular lymphoma who had received two or more lines of prior systemic therapy [32].

The study found that odronextamab achieved deep and durable responses with a generally manageable safety profile. At 20.1 months’ efficacy follow-up, the objective response rate was 80.0%, and the complete response rate was 73.4%. The median duration of complete response was 25.1 months. The median progression-free survival was 20.7 months, and the median overall survival was not reached.

Regarding safety, the most common treatment-emergent adverse events were cytokine release syndrome at 56% (Grade ≥ 3 in 1.7%), neutropenia at 39%, and pyrexia at 38%. Step-up dosing in cycle 1 was employed to help mitigate the risk of CRS. Discontinuation of odronextamab due to adverse events occurred in 16% of patients. The study concludes that odronextamab shows high complete response rates and manageable safety in heavily pretreated patients with R/R follicular lymphoma.

**Table 2:** A comparative table of available bispecific antibodies for follicular lymphoma.

Bispecific Antibody	Study Name/ Design	Patient Population	ORR	CRR	Median Follow-Up	Key Safety Findings
Mosunetuzumab [29] a CD3 × CD20 bispecific antibody	Phase 2, single-arm	R/R FL with ≥2 prior lines, including anti-CD20 and alkylating agent	High objective responses (specific ORR not given, but CRR is 60.0%)	60.0%	18.3 months	Favorable safety profile. CRS: 44% (G1-2: 42%, G3: 1%, G4: 1%). No treatment-related Grade 5 AEs.
Epcoritamab [24] a CD3 × CD20 bispecific antibody	EPCORE NHL-1, Phase 1–2, multicohort, single-arm	Multiply R/R CD20+ FL with ≥2 prior lines of therapy	82.0% (105/128)	63%	Relatively short; additional follow-up planned	Manageable safety profile. Neutropenia: 25% (G3-4). CRS: 65% (G1-2), 2% (G3). ICANS: 6%. Optimized dosing reduced CRS to 49% (no G3/4).
Odronextamab [32] a CD3 × CD20 bispecific antibody	ELM-2 Phase II study	R/R FL after ≥2 prior lines of systemic therapy	80.0%	73.4%	20.1 months	Generally manageable safety profile. CRS: 56% (G ≥ 3: 1.7%). Neutropenia: 39%. Pyrexia: 38%.

A recently published paper [33] presented an analysis comparing epcoritamab with chemoimmunotherapy, mosunetuzumab, and odronextamab for R/R FL after two or more prior systemic therapies. The study, which used matching-adjusted indirect comparison (MAIC), found that epcoritamab significantly improved response rates and survival outcomes compared to chemoimmunotherapy. Specifically, epcoritamab demonstrated higher objective response rates (90.9% vs. 56.8%) and complete response rates (73.7% vs. 32.0%) against chemoimmunotherapy, along with improved progression-free and overall survival. When compared to other bispecific antibodies like mosunetuzumab and odronextamab, epcoritamab showed numerically higher objective response rates and a more favorable safety profile, including lower incidences of severe cytokine release syndrome, neurotoxicity, neutropenia, and infections. The analysis concludes that epcoritamab offers enhanced efficacy and safety in the R/R FL treatment landscape.

### 5.1. Impact of Prior Bispecific Antibody Treatment on CAR-T Outcome

Both CD19 and CD20 are expressed on FL B-cells, but they have distinct expression patterns and histories of therapeutic use that influence why one is often chosen over the other for a specific type of immunotherapy. The bispecific antibodies like epcoritamab, mosunetuzumab, and odronextamab are designed to bind CD20 on the lymphoma cell and CD3 on the patient’s own T-cells, thus redirecting the T-cells to kill the malignant B-cells. CD20 is typically expressed at a very high density on B-cell lymphoma cells (including FL). This high density makes it an excellent target for antibodies, as demonstrated by the success of the initial anti-CD20 monoclonal antibody, rituximab. The high density may be particularly advantageous for T-cell engagement strategies like BsAbs, where proximity is key to activating the T-cell.

CAR T-cells are typically engineered to target CD19 in FL. While CD20 density is higher, CD19 expression is often more homogenous across the B-cell lineage and less prone to loss after previous anti-CD20 therapies (like rituximab). Since CAR T-cell therapy is typically used in the relapsed/refractory (R/R) setting, where patients have often received multiple lines of anti-CD20 therapy, targeting an antigen less likely to be lost due to prior treatment is critical to prevent tumor escape. Ultimately, the differing targets represent an opportunity for sequential therapy. If a patient relapses after CD19 CAR T-cell therapy, they may still respond to a CD20-targeted therapy like a bispecific antibody, or vice versa, offering more treatment options for R/R disease [11].

### 5.2. Comparing MAIC Analyses: Lisocabtagene Maraleucel and Axicabtagene Ciloleucel Versus Mosunetuzumab, and Cost-Effectiveness

Two distinct MAIC analyses shed light on the comparative efficacy and safety of CAR T-cell therapies (lisocabtagene maraleucel and axicabtagene ciloleucel) against mosunetuzumab, a bispecific antibody, in the treatment of relapsed or refractory follicular lymphoma. In the absence of direct head-to-head trials, these analyses provide valuable insights by adjusting for differences in patient populations.

The first MAIC [34] focused on lisocabtagene maraleucel (liso-cel) versus mosunetuzumab for third-line or later (3L+) R/R FL. This analysis, using individual patient data for liso-cel and aggregate data for mosunetuzumab (GO29781 study), indicated that liso-cel was associated with improved efficacy. Specifically, liso-cel demonstrated a higher objective response rate (Odds Ratio [OR], 3.78), a higher complete response rate (OR, 6.46), and improved progression-free survival (Hazard Ratio [HR], 0.28) compared to mosunetuzumab. Regarding safety, while liso-cel had a lower incidence of grade ≥3 cytokine release syndrome (OR, 0.45) and Grade 3–4 serious infections (OR, 0.35), it showed a higher incidence of any-grade CRS (OR, 1.86) and any-grade neurological events (OR, 2.16). The authors concluded that liso-cel offered improved efficacy with a favorable benefit-risk profile.

The second MAIC [35] compared axicabtagene ciloleucel (axi-cel) to mosunetuzumab for R/R FL. This analysis used individual patient data from the ZUMA-5 trial for axi-cel and aggregate data from the GO29781 trial for mosunetuzumab. After weighting by propensity scores, axi-cel showed significantly better efficacy outcomes. The objective response rate was 94.0% for axi-cel versus 65.6% for mosunetuzumab, and the complete response rate was 80.0% versus 43.1%, respectively. The 12-month event-free survival rate was 73.1% for axi-cel compared to 49.3% for mosunetuzumab, while the 12-month progression-free survival rate was 73.5% for axi-cel versus 49.4% for mosunetuzumab. Median duration of response, progression-free survival, and overall survival were not reached for axi-cel, indicating more durable responses, whereas for mosunetuzumab, these medians were 22.8 months, 12.0 months, and not reached, respectively. Overall, axi-cel demonstrated superior efficacy across multiple endpoints compared to mosunetuzumab in this adjusted comparison.

When comparing these two MAIC analyses, both liso-cel and axi-cel, as CAR T-cell therapies, appear to offer superior efficacy outcomes (higher response rates, better progression-free survival) compared to mosunetuzumab. While the specific odds ratios and hazard ratios differ between the liso-cel and axi-cel comparisons, the general trend indicates a more profound and durable response with CAR T-cell therapies.

From a cost-effectiveness perspective, a separate analysis evaluated axicabtagene ciloleucel versus mosunetuzumab in relapsed/refractory follicular lymphoma from a US third-party payer perspective [36]. This study found that axi-cel was associated with increases of 1.51 life years and 1.80 quality-adjusted life years compared to mosunetuzumab. Although axi-cel has a high upfront cost, its total incremental costs were calculated at 151,425, primarily due to the one-time treatment. The incremental cost-effectiveness ratio for axi-cel versus mosunetuzumab was USD 84,016 per quality-adjusted life year gained [36]. This ICER is considered considerably lower than the commonly accepted US willingness-to-pay threshold of USD 150,000 per QALY, suggesting that axi-cel is a cost-effective option [36].

In conclusion, while bispecific antibodies like mosunetuzumab offer a valuable treatment, CAR T-cell therapies such as liso-cel and axi-cel appear to demonstrate superior efficacy in these indirect comparisons. The cost-effectiveness analysis further suggests that despite their higher initial price, CAR T-cell therapies can be a cost-effective long-term solution due to their potential for more durable responses and reduced need for subsequent treatments, leading to an overall improved quality of life. This implies that the higher upfront investment in CAR T-cell therapy may be justified by the long-term benefits and economic value it provides.

## 6. Comparing the Pros and Cons of Bispecific Antibodies and CART Therapy in Follicular Lymphoma

Both bispecific antibodies and CAR T-cell therapy have distinct differences in their administration, safety profiles, and long-term outcomes (Table 3). Bispecific antibodies are generally considered a more accessible, “off-the-shelf” treatment, while CAR T-cell therapy offers the potential for long-term remission or cure with a single infusion, but at the cost of a more intensive treatment process and higher risk of severe side effects.

Bispecific antibodies are “off-the-shelf” products that are readily available for infusion. This avoids the manufacturing and wait times associated with CAR T-cell therapy, which can be crucial for patients with aggressive disease. These therapies are typically administered as outpatient infusions, with a “step-up” dosing schedule during the initial cycle to manage side effects. This is a less burdensome process compared to the required hospitalization for CAR T-cell therapy. While both therapies have overlapping toxicities, particularly cytokine release syndrome (CRS) and neurological events (ICANS), these side effects are generally less severe and more predictable with bispecific antibodies [37].

On the other hand bispecific antibodies require repeated infusions, often for a fixed duration of several months or more, to maintain their therapeutic effect. This contrasts with the single-infusion approach of CAR T-cell therapy. Although highly effective, studies have shown that bispecific antibodies may have slightly lower complete response rates compared to some CAR T-cell therapies. While responses can be durable, the long-term remission and survival data for bispecific antibodies are still emerging, and may not yet match the long-term, potentially curative potential seen with CAR T-cells in some patients [38].

CAR T-cell therapy offers a chance for deep and durable remission after a single infusion. Some long-term data suggest a potential for a cure in a subset of patients. The “one-and-done” nature of the infusion is a significant advantage, as it removes the need for ongoing treatment cycles once the initial recovery period is over. Clinical trials have demonstrated high objective response rates and complete response rates for CAR T-cells in follicular lymphoma, often higher than those seen in initial bispecific antibody studies [39].

However, the manufacturing process for CAR T-cells is complex. It involves collecting a patient’s T-cells, shipping them to a specialized facility for genetic modification, and then shipping them back for infusion. This can take several weeks, during which a patient’s disease could progress. CAR T-cell therapy is associated with a higher risk of severe CRS and neurotoxicity (ICANS). Patients require a mandatory inpatient stay, often in a specialized medical center, for close monitoring and management of these potentially life-threatening side effects. CAR T-cell therapies have a very high upfront cost, though this may be offset by the potential for long-term remission and a reduction in the need for subsequent treatments [40].

## 7. Conclusions

**Figure 1:**
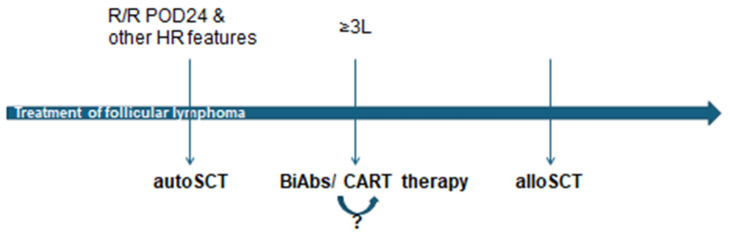
Illustrates the proposed sequence of cellular therapies in follicular lymphoma based on the data previously presented and is the author’s own expert opinion. R/R—relapsed/refractory, POD24—progression of disease within 24 months, HR—high risk, autoSCT—autologous stem cell transplantation, 3L—third line, BiAbs—bispecific antibodies, CART—chimera antigen receptor T-cell, alloSCT—allogeneic stem cell transplantation, ?—uncertainty regarding sequence.

The sequencing of cellular therapies in follicular lymphoma management is a complex and evolving field (Figure 1). AutoSCT remains a valuable option for chemosensitive relapses, while alloSCT is reserved for high-risk cases, like relapsing after bispecific antibodies or CAR-T cell therapy. Also, a high-risk patient responding to bispecific antibodies could be consolidated with alloSCT. CAR-T cell therapy has emerged as a crucial intervention, potentially positioned between autoSCT and alloSCT, maybe even replacing it in the case of some patients. Unfortunately, comparative studies of transplant and novel cellular therapies like CART are not yet available [11]. The optimal use of bispecific antibodies and their impact on subsequent CART cell therapy also requires further investigation. However, bispecific antibodies are generally favored over CAR T-cell therapy as a potential third-line treatment for patients with R/R FL who do not present with aggressive histologic transformation. This consideration is informed by the 3-year follow-up of the mosunetuzumab phase 2 trial in R/R FL, highlighting the potential for durable complete responses after a time-limited therapy, alongside an acceptable safety profile for patients across a spectrum of ages and performance statuses [30]. In cases of confirmed or suspected histologic transformation, CAR-T cell therapy is often prioritized over bispecific antibodies (BiAbs) given its curative potential, as indicated by trial data from R/R diffuse large B-cell lymphoma; the extent to which BiAbs offer comparable long-term benefits in transformed FL remains an open question [8]. Future research should focus on identifying predictive biomarkers and refining treatment algorithms to optimize outcomes in FL.

## Figures and Tables

**Table 1:** A summary and detailed comparison of the three major CAR-T cell trials: ZUMA-5, ELARA, and TRANSCEND FL.

Trial	CAR-T Product	Patient Population	Overall Response Rate	Complete Remission Rate	Key Findings
ZUMA-5 [25]	Axicabtagene Ciloleucel (axi-cel) directed against the CD19 antigen	Relapsed/Refractory (R/R) indolent Non-Hodgkin Lymphoma (iNHL), including follicular lymphoma and marginal zone lymphoma, after ≥2 lines of therapy	94% (Primary analysis: 92%)	80% (Primary analysis: 74%)	Demonstrated continued durable responses after 3 years, with very few relapses beyond 2 years. Median progression-free survival in FL was 40.2 months. Elevated baseline total metabolic tumor volume and recent prior bendamustine use may affect durable remissions.
ELARA [26]	Tisagenlecleucel directed against the CD19 antigen	R/R Follicular Lymphoma (grades 1–3A) after ≥2 lines of prior therapy or after autologous stem cell transplantation (auto-SCT)	86%	69%	Showed highly durable efficacy and a favorable safety profile with a median follow-up of 29 months 24-month PFS rate was 57.4%. No new safety signals or treatment-related deaths were reported. Low levels of tumor-infiltrating LAG3 + CD3+ exhausted T cells and higher baseline levels of naive CD8+ T cells were associated with improved outcomes.
TRANSCEND FL [27]	Lisocabtagene Maraleucel (liso-cel) directed against the CD19 antigen	R/R Follicular Lymphoma, including second-line (2L) patients who all had progression of disease within 24 months from diagnosis % (L+), 96% (2L)	97%	94% (3L+), 100% (2L)	Showed promising results for both 2L and 3L+ R/R FL; minimal residual disease negativity was observed. Cytokine release syndrome occurred in 58% of patients (Grade ≥ 3, 1%); neurological events occurred in 15% of patients (Grade ≥ 3, 2%). The patient population was relatively young, consistent with other phase 2 studies for axi-cel and tisagenlecleucel.

**Table 3:** Comparing the pros and cons of bispecific antibodies and CART therapy in follicular lymphoma.

Bispecific Antibodies	CART Therapy
off the shelf	currently approved products are autologous (require apheresis)
no risk of manufacturing failure	potential for manufacturing failures
no lymphodepletion	requires lymphodepletion
lower incidence and severity of CRS/ICANS	increased incidence of high-grade CRS/ICANS
requires multiple doses	one and done administration
high incidence of infections, need for IVIG support	high incidence of infections, need for IVIG support
dose interruption allowed to manage toxicities	

## Data Availability

Not applicable.
